# Increasing the Ascomycin Yield by Relieving the Inhibition of Acetyl/Propionyl-CoA Carboxylase by the Signal Transduction Protein GlnB

**DOI:** 10.3389/fmicb.2021.684193

**Published:** 2021-05-26

**Authors:** Pan Wang, Xin Wang, Ying Yin, Mingliang He, Wei Tan, Wenting Gao, Jianping Wen

**Affiliations:** ^1^Key Laboratory of Systems Bioengineering (Ministry of Education), Tianjin University, Tianjin, China; ^2^SynBio Research Platform, Collaborative Innovation Center of Chemical Science and Engineering (Tianjin), School of Chemical Engineering and Technology, Tianjin University, Tianjin, China; ^3^Frontiers Science Center for Synthetic Biology (Ministry of Education), Tianjin University, Tianjin, China

**Keywords:** PII signal transduction protein, acetyl-CoA carboxylase, propionyl-CoA carboxylase, GlnB, ascomycin

## Abstract

Ascomycin (FK520) is a multifunctional antibiotic produced by *Streptomyces hygroscopicus* var. *ascomyceticus*. In this study, we demonstrated that the inactivation of GlnB, a signal transduction protein belonging to the PII family, can increase the production of ascomycin by strengthening the supply of the precursors malonyl-CoA and methylmalonyl-CoA, which are produced by acetyl-CoA carboxylase and propionyl-CoA carboxylase, respectively. Bioinformatics analysis showed that *Streptomyces hygroscopicus* var. *ascomyceticus* contains two PII family signal transduction proteins, GlnB and GlnK. Protein co-precipitation experiments demonstrated that GlnB protein could bind to the α subunit of acetyl-CoA carboxylase, and this binding could be disassociated by a sufficient concentration of 2-oxoglutarate. Coupled enzyme activity assays further revealed that the interaction between GlnB protein and the α subunit inhibited both the activity of acetyl-CoA carboxylase and propionyl-CoA carboxylase, and this inhibition could be relieved by 2-oxoglutarate in a concentration-dependent manner. Because GlnK protein can act redundantly to maintain metabolic homeostasis under the control of the global nitrogen regulator GlnR, the deletion of GlnB protein enhanced the supply of malonyl-CoA and methylmalonyl-CoA by restoring the activity of acetyl-CoA carboxylase and propionyl-CoA carboxylase, thereby improving the production of ascomycin to 390 ± 10 mg/L. On this basis, the co-overexpression of the β and ε subunits of propionyl-CoA carboxylase further increased the ascomycin yield to 550 ± 20 mg/L, which was 1.9-fold higher than that of the parent strain FS35 (287 ± 9 mg/L). Taken together, this study provides a novel strategy to increase the production of ascomycin, providing a reference for improving the yield of other antibiotics.

## Introduction

Ascomycin (FK520) is a natural 23-membered macrocyclic antibiotic produced by *Streptomyces hygroscopicus* var. *ascomyceticus* ATCC 14891 (Wu et al., 2000), notable for its diverse biological and pharmacological activities, including antifungal (Qi et al., 2014a), antimalarial (Monaghan et al., 2005), immunosuppressive (Dumont et al., 1992), and anticonvulsive effects (Sierra-Paredes and Sierra-Marcuno, 2008). Consequently, it has been widely used in the clinical treatment of autoimmune diseases, skin diseases and organ transplant rejections (Qi et al., 2014a, b; Song et al., 2017).

Due to the growing market demand and broad application prospects of FK520, many studies attempted to improve the productivity of engineered strains through various methods. For example, based on metabolic profiling analysis, the exogenous feeding of 2% *n*-hexadecane at 24 h, 1.0 g/L valine at 48 h and 1.0 g/L lysine at 96 h improved the production of FK520 to 460 mg/L, which was 53.3% higher than without exogenous feeding (Qi et al., 2014a). The co-overexpression of the pathway-specific regulatory gene *fkbR1* and its target gene *fkbE* increased the yield of FK520 to 536.7 mg/L, which was 69.9% higher than that of the parent strain *S. hygroscopicus* var. *ascomyceticus* FS35 (Song et al., 2017). In addition, the selection of shikimic acid-resistant strain *S. hygroscopicus* var. *ascomyceticus* SA68 and the addition of 3 g/L shikimic acid at 24 h increased the production of FK520 to 450 mg/L, which was 53.3% higher than in the initial strain FS35 (Qi et al., 2014b). In spite of these advances, the insufficient supply of precursors for the secondary metabolite FK520 is still a pressing problem limiting its yield.

**FIGURE 1:**
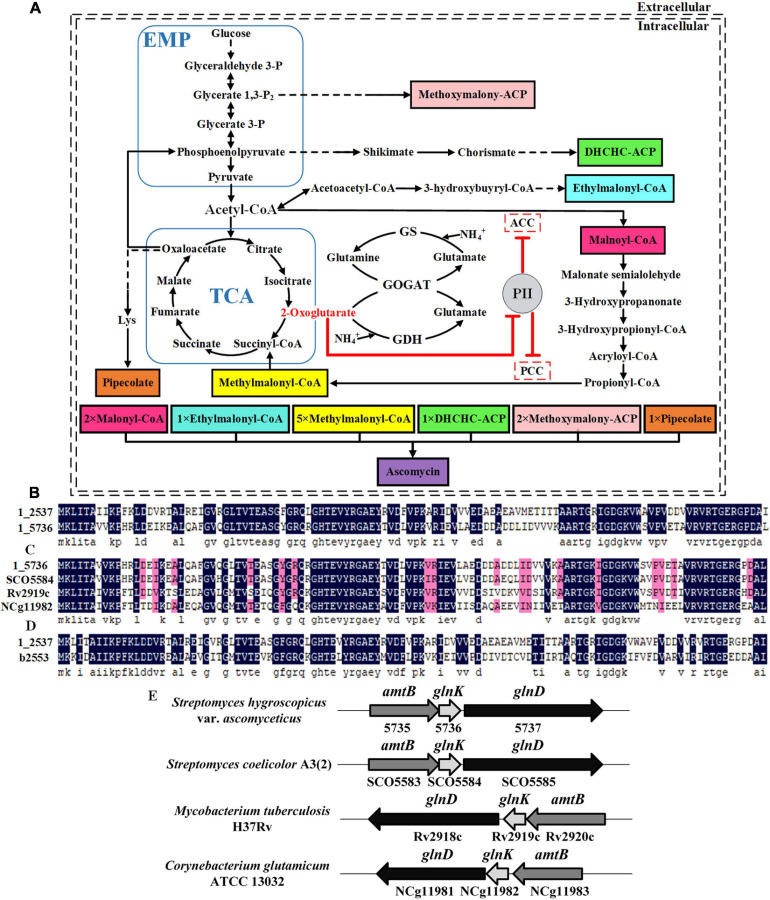
Identification of two PII family signal transduction proteins in *Streptomyces hygroscopicus* var. *ascomyceticus* and their influence on the biosynthesis of FK520 precursors. **(A)** A schematic view of the major primary metabolic pathways for the synthesis of FK520 precursors and the regulation of precursors malonyl-CoA and methylmalonyl-CoA biosynthesis by PII family signal transduction proteins. The solid black lines represent one-step reactions and the dotted black lines represent multi-step reactions. Shaded boxes in different colors represent different precursors of ascomycin. The dotted boxes represent enzymes regulated by the PII family signal transduction proteins. The arrows with flat T ends indicate the inhibitory role. **(B)** Amino acid sequence alignment of two PII proteins from *S. hygroscopicus* var. *ascomyceticus* FS35. The amino acid sequences of these two PII proteins have been uploaded to the GeneBank database (1_2537: MW936359, 1_5736: MW936360). **(C)** Amino acid sequence alignment between the PII protein encoded by the 1_5736 gene from *S. hygroscopicus* var. *ascomyceticus* FS35 and the GlnK proteins from other actinobacteria. SCO5584 represents the GlnK protein from *Streptomyces coelicolor* A3(2), which is retrieved from the NCBI database (TYP02429.1). Rv2919c represents the GlnK protein from *Mycobacterium tuberculosis* H37Rv, which is retrieved from the NCBI database (AFN50901.1). NCg11982 represents the GlnK protein from *Corynebacterium glutamicum* ATCC13032, which is retrieved from the NCBI database (BAB99453.1). **(D)** Amino acid sequence alignment between the PII protein encoded by the 1_2537 gene from *S. hygroscopicus* var. *ascomyceticus* FS35and the GlnB protein of *Escherichia coli* K-12. The amino acid sequence of the GlnB protein in *E. coli* K-12 was retrieved from the NCBI database (AAC75606.1). All the sequence alignments were performed using DNAMAN (Version6). The dark blue shadows highlight the identical residues and the residues with more than 75% sequence similarity are shaded in pink. **(E)** Arrangement of the *amtB-glnK-glnD* operon in different actinobacteria. The shaded arrow boxes represent different genes and the direction of each arrow represents the direction of transcription. The length of boxes is proportional to the length of each gene sequence.

In *S. hygroscopicus* var. *ascomyceticus*, FK520 is assembled from 12 precursor molecules (Mo et al., 2012b), with the majority being malonyl-CoA (2 molecules) and methylmalonyl-CoA (5 molecules) (Figure 1A). Thus, the biosynthesis of malonyl-CoA and methylmalonyl-CoA is crucial for the production of FK520. *In vivo*, acetyl-CoA carboxylase (ACC) catalyzes the conversion of acetyl-CoA into malonyl-CoA, while propionyl-CoA carboxylase (PCC) catalyzes the conversion of propionyl-CoA into methylmalonyl-CoA (Figure 1A). Stable overexpression of ACC in *Streptomyces coelicolor* was reported to effectively promote the biosynthesis of actinorhodin, which uses malonyl-CoA as one of the precursors (Ryu et al., 2006; Li et al., 2019). The overexpression of ACC in *Actinomadura hibisca* P157-2 was also found to improve the production of actinorhodin (Paudel et al., 2011). Similarly, the heterologous expression of PCC in *Streptomyces* sp. RM7011 significantly increased the yield of tacrolimus (FK506), which uses methylmalonyl-CoA as one of the precursors (Mo et al., 2009, 2012a).

Acetyl-CoA carboxylase (ACC) and propionyl-CoA carboxylase (PCC) are biotin-dependent carboxylases, composed of three functional units: the biotin carboxylase (BC), the biotin carboxyl carrier protein (BCCP), and the carboxyl transferase (CT) (Tong, 2013). The entire carboxylation reaction catalyzed by ACC or PCC consists of two steps. Firstly, BC attaches a carbonate to the biotin prosthetic group of BCCP to form carboxybiotin, a reaction that depends on the hydrolysis of ATP. Secondly, CT transfers the carboxyl from biotin to acyl-CoA to produce the corresponding carboxylated acyl-CoA (Oh et al., 2006; Lyonnet et al., 2017). In *Escherichia coli*, *Azospirillum brasilense* and *Synechocystis* sp. PCC6803, the activity of ACC was shown to be regulated by signal transduction proteins belonging to the PII family, which respond to the intracellular concentrations of effector molecules such as ATP, ADP and 2-oxoglutarate (2-OG) (Gerhardt et al., 2015; Hauf et al., 2016).

PII proteins are crucial signal transduction molecules that are widely distributed in bacteria, archaea, and plants, where they coordinate many facets of nitrogen metabolism (Fokina et al., 2010; Radchenko et al., 2013). By responding to intermediate metabolites and the abundance of carbon and nitrogen, PII proteins can flexibly regulate the activity of key enzymes [glutamine synthetase (GS), N-acetyl-l-glutamate kinase (NAGK), and nitrogenase], membrane transport proteins, and transcription factors (NtrB/NtrC, NtcA, TnrA, AmtR, and NifA), thereby maintaining the intracellular balance between carbon and nitrogen metabolism (Huergo et al., 2013). In recent years, studies have found that some ACC enzymes from plants and bacteria are also regulated by PII proteins (Forchhammer and Selim, 2020). In *Arabidopsis thaliana*, *Chlamydomonas reinhardtii*, *A. brasiliense*, and *E. coli*, PII proteins can inhibit the activity of ACC by interacting with the BCCP functional unit, and this inhibition can be relieved by a sufficiently high concentration of 2-OG (Bourrellier et al., 2010; Rodrigues et al., 2014; Gerhardt et al., 2015). Previous studies showed that the deletion of PII proteins dysregulates ACC activity, leading to an increase of lipid bodies, which use the product of ACC as a starting unit (Zalutskaya et al., 2015; Hauf et al., 2016).

In actinobacteria, both ACC and PCC are complex multi-subunit enzymes. They share the same α subunit composed of BC and BCCP functional domains, but possess a specific β subunit for substrate recognition and a specific ε subunit for protein-protein interactions (Diacovich et al., 2002; Lyonnet et al., 2017). Furthermore, both ACC and PCC catalyze similar two-step carboxylation reactions (Oh et al., 2006), and their catalytic substrates even overlap to a certain extent (Diacovich et al., 2002). Accordingly, it was speculated that ACC and PCC might both be regulated by PII proteins in actinobacteria (Figure 1A). Since the carboxylation products of these two enzymes, malonyl-CoA and methylmalonyl-CoA, are important precursors for the biosynthesis of polyketide antibiotics (Rodriguez et al., 2001; Demirev et al., 2011), it is of great significance to explore the influence of PII proteins on the activity of ACC and PCC, with the aim of improving the antibiotic yield.

In this study, the effect of PII proteins on the activity of ACC and PCC in *S. hygroscopicus* var. *ascomyceticus* was investigated to develop new strategies for the improvement of FK520 production. Firstly, two native PII signal transduction proteins of *S. hygroscopicus* var. *ascomyceticus* were identified through bioinformatic analysis. Then, protein co-precipitation was performed to determine whether there is an interaction between the purified PII proteins and the α subunit of ACC. The effect of PII proteins on the *in vitro* activity of ACC and PCC was assessed using coupled enzyme activity assays. The influence of PII proteins on the intracellular activity of ACC and PCC was inferred from the changes in the concentrations of coenzyme A esters caused by PII deletion. On this basis, rational gene engineering strategies were proposed to increase the yield of FK520.

## Materials and Methods

### Strains, Plasmids, and Growth Conditions

The parent strain used in this study, *S. hygroscopicus* var. *ascomyceticus* FS35, was selected from *S. hygroscopicus* var. *ascomyceticus* ATCC 14891 after femtosecond laser irradiation (Qi et al., 2014a). All the strains and plasmids used in this study are listed in Supplementary Table 1. Strain FS35 and its derivatives were cultured and passaged on MS solid medium (20 g/L soybean cake meal, 20 g/L mannitol, and 20 g/L agar) at 28∘C. When the colonies on MS medium produced black spores, the spores were used to inoculate liquid seed medium and growth for 60 h at 28∘C and 220 rpm. The composition of the seed medium was same as described previously (Qi et al., 2014b). Then, the seed cultures were transferred into fermentation medium at an inoculation ratio of 10%. The fermentation broth was incubated for 192 h at 28∘C and 220 rpm. The fermentation medium used for the measurement of various fermentation parameters contained 20 g/L soluble starch, 40 g/L dextrin, 5 g/L yeast powder, 5 g/L peptone, 5 g/L corn steep liquor, 1 g/L K_2_HPO_4_, 1.5 g/L (NH_4_)_2_SO_4_, 0.5 g/L MnSO_4_, 1 g/L MgSO_4_⋅7H_2_O, 1 g/L CaCO_3_, and 2.5 mL/L soybean oil. *E. coli* DH5α was used for plasmid construction. *E. coli* ET12567 (pUZ8002) was used as the donor strain for intergeneric conjugation with S. *hygroscopicus* var. *ascomyceticus*. *E. coli* BL21(DE3) was used for heterologous protein expression. All *E. coli* strains were cultured in Luria-Bertani (LB) medium at 37∘C. The plasmid pET28a(+) was used to construct the protein expression vector. The plasmid pKC1139 was used to construct the gene deletion vector. The plasmid pSET152 was used for the construction of a gene complementation vector. The plasmid pIB139, which contains the strong promoter *ermE^∗^p*, was used for the construction of a gene overexpression vector.

### Gene Deletion, Complementation, and Overexpression

Transformation of *Streptomyces* was done using intergeneric conjugation with an *E. coli* donor strain according to standard methods (Kieser et al., 2000; Ma et al., 2018). The raw genome sequences were uploaded to the Gene Expression Omnibus (GEO) database at the National Center for Biotechnology Information (NCBI) (accession number: GSE143832). All primers used for gene manipulation are listed in Supplementary Table 2. To construct the *glnB* deletion strain Δ*glnB*, the 1,009-bp upstream flanking region and 1,003-bp downstream flanking region of *glnB* were, respectively, amplified from the genome of FS35 using the primer pairs DglnB-UF/DglnB-UR and DglnB-DF/DglnB-DR. The obtained fragments were fused by overlap-extension PCR using the primer pair DglnB-UF/DglnB-DR. The fusion fragment was inserted into the plasmid pKC1139 between the *Hin*dIII and *Xba*I sites to obtain the *glnB* deletion vector pKCglnB. The recombinant plasmid pKCglnB was introduced into FS35 by traditional intergeneric conjugation. The single-crossover transformants were selected on MS solid medium containing 50 μg/mL apramycin. After two rounds of sporulation on MS solid medium without apramycin, the apramycin-sensitive colony was selected as the double crossover strain. The deletion strain was verified by PCR and sequencing using the primer pair VDglnB-F/VDglnB-R. The *glnK* deletion strain Δ*glnK* and the *glnB*-*glnK* double deletion strain Δ*glnB*Δ*glnK* were constructed using the same method, and were verified by PCR and sequencing using the primer pairs VDglnB-F/VDglnB-R and VDglnK-F/VDglnK-R, respectively.

For the construction of the *glnK* complementation strain Δ*glnB*Δ*glnK*/pSETglnK based on the double deletion strain Δ*glnB*Δ*glnK*, the native promoter sequence of the *amtB-glnK-glnD* operon was amplified from the genome of FS35 using the primer pair CglnK-PF/CglnK-PR. The DNA sequence of *glnK* was amplified from the genome of FS35 using the primer pair CglnK-KF/CglnK-KR. The obtained fragments were fused by overlap-extension PCR using the primer pair CglnK-PF/CglnK-KR. The fusion fragment was inserted into the vector pSET152 between the *Bam*HI and *Xba*I sites to construct the complementation vector pSETglnK. The recombinant vector pSETglnK was introduced into the Δ*glnB*Δ*glnK* strain by traditional intergeneric conjugation to obtain the complementation strain Δ*glnB*Δ*glnK*/pSETglnK. which was verified by PCR and sequencing using the primer pair pSET152-F/pSET152-R.

For the construction of the *pccB* overexpression strain Δ*glnB*/pIBOpccB based on the Δ*glnB* strain, the DNA sequences of *pccB* was amplified from the genome of FS35 using the primer pair OpccB-F/OpccB-R. The obtained fragment was inserted into the vector pIB139 between the *Nde*I and *Xba*I sites to construct the overexpression vector pIBOpccB, in which the *pccB* coding sequence was placed under the control of the strong constitutive promoter *ermE^∗^p*. The recombinant vector pIBOpccB was introduced into the Δ*glnB* strain by traditional intergeneric conjugation to obtain the overexpression strain Δ*glnB*/pIBOpccB, which was verified by PCR and sequencing using the primer pair pIB139-F/pIB139-R. The *pccE* overexpression strain Δ*glnB*/pIBOpccE and the *pccB*-*pccE* co-overexpression strain Δ*glnB*/pIBOpccBE were constructed based on the Δ*glnB* strain using the same method. They were verified by PCR and sequencing using the primer pair pIB139-F/pIB139-R.

### Measurements of FK520, FK523, and Biomass Concentrations

To measure the production of FK520 and FK523, 2 ml of fermentation broth was mixed with 3 ml of ethanol. After 30 min of ultrasonic extraction, and 10 min of centrifugation at 8,000 × g, the supernatant was filtered through a 0.2 μm syringe-driven filter (Spartan10463100, Whatman, England), suitable for organic solvents. The concentrations of FK520 and FK523 were quantified by liquid chromatography on a 1100 series instrument (Agilent, United States), equipped with a C-18 column (150 mm × 4.6 mm, 3.5 μm; Agilent). The mobile phase and gradient elution program were same as reported previously (Yu et al., 2019). The flow rate was 2 mL/min and the detection wavelength was 205 nm. The injection volume was 20 μL and the column temperature was 60∘C.

To measure the biomass concentration, mycelia from 5 ml of fermentation broth were washed once with 0.1 M-HCl solution and twice with Milli-Q water. After centrifugation for 10 min at 8,000 × *g*, the wet cell pellet was dried in an oven at 80∘C until a constant weight to measure the dry cell weight (DCW). The biomass concentration was defined as the ratio of DCW to the volume of the fermentation broth.

### Expression and Purification of Proteins

In order to obtain the native or His_6_-tagged proteins, the target proteins were overexpressed in *E. coli* BL21 (DE3). All primers used for heterologous protein expression are listed in Supplementary Table 3. The nucleotide sequences of target proteins were amplified from the genome of FS35 using the corresponding primers. These resulting DNA fragments were digested with the listed restriction enzymes (Supplementary Table 3), and inserted into the corresponding sites of the pET28a (+) vector through enzymatic ligation. The constructed recombinant plasmids were introduced into *E. coli* BL21 (DE3) for the heterologous expression of target proteins. All recombinant strains were cultivated in Luria-Bertani (LB) medium with 50 μg/mL of kanamycin at 37∘C until the optical density of the fermentation broth at 600 nm (OD_600_) reached 0.6–0.7, after which 0.5 mM of Isopropyl β-d-thiogalactoside (IPTG) was added to induce the expression of target proteins. After 3 h of expression at 37∘C, the cells were collected by centrifugation at 12,000 × *g* for 10 min and resuspended in lysis buffer (50 mM Tris-HCl, 0.1 M KCl, and 20% glycerol, pH 7.5). The cells were then disrupted by sonication, the lysate was centrifuged at 12,000 × *g* for 10 min to remove cell debris, and the resulting cleared supernatant was collected for protein purification. All α subunits used in this study were completely biotinylated according to a published method before purification (Rodrigues et al., 2014).

The His_6_-tagged proteins were purified using a Ni^2+^-nitrilotriacetic acid (Ni^2+^-NTA) agarose affinity chromatography column (Qiagen, Germany), according to the manufacturer’s protocol. The native proteins were purified using a Hi-Trap Heparin column (GE Healthcare), which was pre-equilibrated with a previously published buffer (Moure et al., 2012). The proteins were eluted with a buffer gradient from 0.1 to 1 M KCl. After further dialyzed in a previously published buffer (Rodrigues et al., 2014), the eluted proteins were verified by SDS-polyacrylamide gel electrophoresis (SDS-PAGE) (Supplementary Figure 1). The concentration of the purified proteins was measured using a NanoDrop 2000 spectrophotometer (Thermo Fisher Scientific, United States). The purified proteins were stored at −80∘C for subsequent protein co-precipitation and electrophoretic mobility shift assays (EMSA).

### Protein Co-precipitation

The formation of complexes between proteins was assessed using *in vitro* protein co-precipitation as described previously (Huergo et al., 2007). Briefly, 5 μL of His-magnetic beads were washes twice with buffer containing 50 mM Tris-HCl pH 8, 0.1 M NaCl, 0.1% (w/v) lauryl dimethyl amine oxide (LDAO), 20 mM imidazole, 10% glycerol and 5 mM MgCl_2_, together with the indicated effectors (2-OG and ATP) to achieve the pre-equilibrium state. The purified proteins (20 μg GlnB, 20 μg His-GlnB, 20 μg GlnK, 20 μg α subunit, 20 μg His-α subunit) required for the different experiments were added into 500 μL of buffer to induce complex formation. After binding for 20 min, the magnetic beads were washed three times with the binding buffer. The washed beads were then incubated in 20 μL of buffer containing 0.5 M imidazole for 5 min, and the resulting eluent was analyzed by 15% acrylamide SDS-PAGE followed by staining Coomassie brilliant blue R-250 to determine the components of the protein complex.

### Measurement of ACC/PCC Activity *in vitro*

The *in vitro* activity of ACC/PCC was measured using previously described coupled enzyme activity assay (Beez et al., 2009; Gerhardt et al., 2015), with some modifications. The hydrolysis of ATP catalyzed by ACC was coupled to the formation of pyruvate catalyzed by pyruvate kinase (PK) and then coupled to the oxidation of NADH catalyzed by the lactate dehydrogenase (LDH). The coupled enzymatic reactions were carried out in buffer containing 10 mM NaHCO_3_, 10 mM ATP, 1 mM phosphoenolpyruvate, 0.2 mM NADH, 20 mM MgCl_2_, 50 mM KCl, 0.5 mM dithiothreitol (DTT), 50 mM imidazole, 4.4 units of LDH, and 6 units of PK. The reaction systems also included 10 nM AccB or 10 nM PccB, 20 nM AccA, along with the indicated concentrations of PII proteins and 2-OG, in a final volume of 400 μL with a final pH of 7.5. After the reaction systems were pre-incubated at 25∘C for 15 min, 400 μM acetyl-CoA or 400 μM propionyl-CoA was added to start the enzymatic reactions. The oxidation of NADH over a period of 20 min at 25∘C was measured by recording the decrease at 340 nm using a SPECORD 200 photometer (Analytik, Jena).

### Measurement of Intracellular Coenzyme A Esters

In order to measure the content of intracellular coenzyme A esters, mycelia obtained form 5 mL of fermentation broth were washed twice with deionized water and centrifuged at 8,000 × *g* for 10 min. The wet hyphae were suspended in 300 μL of 15% trichloroacetic acid and lysed by vortexing for 3 min at 4∘C with 150 μL of glass beads. After centrifugation for 10 min at 8,000 × *g* and 4°C, the supernatant was passed through an OASIS HLB SPE cartridge (waters, United States) under vacuum to extract coenzyme A esters, as reported previously (Mo et al., 2012a). The analysis of coenzyme A esters was performed using Ultra-high performance liquid chromatography (UPLC, Waters, United States)-electrospray ionization (ESI)-tandem mass spectrometry (MS; Xveo TQ-XS, Waters, United States), equipped with an ACQUITY UPLC HSS T3 column (100 mm × 2.1 mm, 1.7 μm; Waters, United States), as previously reported, with some modifications (Park et al., 2007). The mobile phase was composed of 5 mM ammonium acetate and 0.05% acetic acid in water (A), and 80% acetonitrile with the same additive concentration (B). The gradient elution program included: 0–0.8 min, 90% A/10% B; 0.8–2 min, a linear gradient from 90% A/10%B to 60% A/40% B; 2–6 min, a linear gradient from 60% A/40% B to 20% A/80% B; 6–8 min, 20% A/80% B; 8–12 min, and a linear gradient from 20% A/80% B to 90% A/10% B. The quantification was done in multiple reaction monitoring (MRM) mode with two mass ions: *m*/*z* parent > *m*/*z* daughter (acetyl-CoA, 810 > 303; malonyl-CoA, 854 > 347; propionyl-CoA, 824 > 317; methylmalonyl-CoA, 868 > 361).

### Electrophoretic Mobility Shift Assays (EMSA)

For the EMSA, the promoter sequence of the *amtB-glnK-glnD* operon was amplified from the genome of FS35 using the primer pair pBKD-F/pBKD-R (Supplementary Table 3). The Cy5-labeled primer 5′-AGCCAGTGGCGATAAG-3′ was used for the secondary amplification of the obtained promoter sequence to generate the Cy5-labeled DNA probes. Then 5 ng of the labeled probe was incubated for 30 min at 28∘C with different concentration of His_6_-GlnR protein in a previously described reaction buffer (Zhang et al., 2019). After mixing with the loading buffer (0.25 × Tris borate EDTA (TBE) buffer, 60% glycerol), the reaction mixture was loaded on to 6% native polyacrylamide gels and separated for 40 min in an ice bath containing 0.5 × TBE at 90 V. Then, the DNA bands with bound or unbound proteins were visualized by fluorescence imaging using a Typhoon Trio variable mode imager (GE Healthcare, United States).

### Quantitative Real-Time PCR (qRT-PCR) Analysis

The transcriptional levels of gene *glnK* in the parent strain FS35 and the deletion strain Δ*glnB* were measured by qRT-PCR at 60 h. The primers used for qRT-PCR were listed in Supplementary Table 3. Firstly, the fermentation broth of FS35 and Δ*glnB* was collected at 60 h. After centrifugation at 8,000 × g for 10 min, the wet hyphae were rapidly frozen in liquid nitrogen. Then the total RNA was extracted from the frozen hyphae using RNAprep Pure Cell/Bacteria Kit (Tiangen, Beijing, China). The concentration and integrity of the RNA sample was detected by 1% agarose gel electrophoresis. Then the RNA sample was reversely transcribed into cDNA by using PrimeScript^TM^ RT reagent Kit with gDNA Eraser (takara, Japan). To exclude DNA contamination, the RNA sample which treated by gDNA Eraser but not reverse transcription was used as a template for negative control. With the cDNA as template, qRT-PCR was carried out on a LightCycler^®^ 480 using SYBR Green Master Mix (Roche, Switzerland). The 16S rRNA was used as the internal reference gene, and the change folds of the transcriptional levels were quantified relatively with the comparative *C*_*T*_ method (Wang et al., 2018).

### Statistical Analysis

In this study, the samples used for the measurement of biomass and FK520 yield were taken from five independent technical replicates. The samples used for the analysis of coenzyme A esters’ content, enzyme activity and quantitative real-time PCR were taken from three independent technical replicates. All data were presented as the mean values of respective independent technical replicates and the error bars indicate the standard deviations (SD).

## Results

### Identification of Two PII Signal Transduction Proteins in *Streptomyces hygroscopicus*

The signal transduction proteins belonging to the PII family are classified into three subgroups, GlnK, GlnB, and NifI (Huergo et al., 2013). The genome-wide sequencing results revealed that there are two PII signal transduction proteins (1_2537 and 1_5736) in *S. hygroscopicus* var. *ascomyceticus*. The amino acid sequence alignment showed that these two PII signal transduction proteins shared 66.96% amino acid sequence identity (Figure 1B). In order to determine which subgroups these two PII signal transduction proteins belong to, the amino acid sequences encoded by the 1_2537 and 1_5736 genes of *S. hygroscopicus* var. *ascomyceticus* were aligned with the GlnK proteins from three other actinobacteria (*S. coelicolor*, *Mycobacterium tuberculosis*, and *Corynebacterium glutamicum*) and the GlnB protein from *E. coli* (Figures 1C,D). The sequence alignment indicated that the protein encoded by 1_5736 shared approximately 81% amino acid sequence identity with the GlnK proteins of other actinobacteria (Figure 1C), while the protein encoded by 1_2537 shared approximately 61% amino acid identity with the GlnB protein of *E. coli* (Figure 1D). This implied that the protein encoded by 1_2537 belonged to the GlnB subgroup of PII signal transduction proteins, while the protein encoded by 1_5736 belonged to the GlnK subgroup.

The gene encoding GlnK protein in actinobacteria is located adjacent to the *amtB* gene (responsible for ammonia transfer) and the *glnD* gene (responsible for uridylyl transfer) in an *amtB-glnK-glnD* operon (Arcondeguy et al., 2001; Huergo et al., 2013). The genome-wide sequencing results and sequence homology analysis revealed that 1_5735 and 1_5737, the genes adjacent to 1_5736 in *S. hygroscopicus* var. *ascomyceticus*, respectively, encoded the proteins AmtB and GlnD (Supplementary Figure 2). They also formed a complete operon with gene 1_5736, as in other actinobacteria (Figure 1E). This further confirmed that the 1_5736 gene of *S. hygroscopicus* var. *ascomyceticus* belongs to the GlnK subgroup of PII signal transduction proteins.

### Interaction Between the Signal Transduction Protein GlnB and the α Subunit of ACC

**FIGURE 2:**
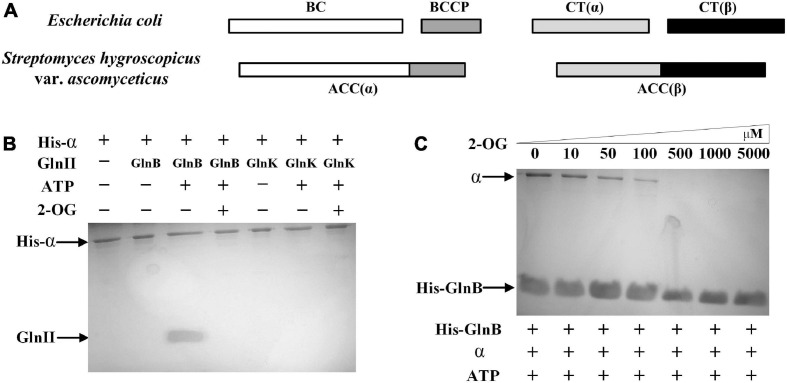
Components of acetyl-CoA carboxylase (ACC) in *Streptomyces hygroscopicus* var. *ascomyceticus* and the interactions of the two PII proteins with α subunit of ACC. **(A)** Comparison of ACC composition between *S. hygroscopicus* var. *ascomyceticus* and *Escherichia coli*. The shaded boxes represent different functional units. The individual shaded boxes indicate different subunits. Connected shaded boxes indicate subunits that are fused into a single polypeptide chain. **(B)** Protein co-precipitation experiments to demonstrate the interaction between the PII proteins and the α subunit of ACC. The reactions were performed in buffer containing 2 mM 2-OG or 3.5 mM ATP. **(C)** The effect of 2-OG on the interaction between GlnB protein and the α subunit of ACC. The reactions were carried out in buffer containing different concentrations of 2-OG and 3.5 mM ATP. The blots on the gel map indicate the proteins eluted in the pull-down experiments. The slope above the blots indicates an increase in the gradient of 2-OG concentration from left to right.

In *E. coli*, the BC and BCCP functional subunits of ACC are, respectively, composed of two different polypeptides (Figure 2A). However, the BC and BCCP functional units of actinobacteria are fused into a single protein, which is called the α subunit of ACC (Tong, 2013). The amino acid sequence alignment between the α subunit of ACC annotated in the genome-wide sequencing and the α subunit of ACC with known biological function from other actinobacteria showed that the 1_3403 gene (*accA*) encoded the α subunit of ACC in *S. hygroscopicus* var. *ascomyceticus* (Supplementary Figure 3). This also confirmed that the BC and BCCP functional units were also fused together to form the α subunit of ACC in *S. hygroscopicus* var. *ascomyceticus*, as in other actinobacteria (Figure 2A).

To assess whether there was a similar interaction between the α subunit of ACC and two PII signal transduction proteins in *S. hygroscopicus* var. *ascomyceticus* as is found in *E. coli*, where the GlnB protein inhibits the activity of ACC by binding with the BC-BCCP complex (Gerhardt et al., 2015), the His-tagged α subunit and two native PII signal transduction proteins were purified and mixed with ATP or 2-OG for protein co-precipitation experiments (Figure 2B). The results of SDS-PAGE analysis showed that the His-tagged α subunit and GlnB protein could be simultaneously eluted by imidazole in the presence of ATP (Figure 2B, lane 3), and this complex disassociated in the presence of a sufficient 2-OG concentration (Figure 2B, lane 4). By contrast, GlnK protein was not found in the eluent irrespective of the presence of ATP or 2-OG (Figure 2B, lane 6 and 7). This suggested that GlnK protein could not bind to the α subunit, so it might not affect the activity of ACC in *S. hygroscopicus* var. *ascomyceticus*.

To further investigate the effects of 2-OG at different concentrations on the interaction between the GlnB protein and the α subunit, protein co-precipitation experiments were carried out with His-tagged GlnB as the bait at different concentrations of 2-OG. The corresponding SDS-PAGE gels showed that when His-tagged GlnB was immobilized on the Ni^2+^ beads as bait, both His-tagged GlnB protein and the α subunit could be eluted in the presence of ATP (Figure 2C, lane 1). This in turn confirmed the binding of GlnB protein to the α subunit. In addition, when the concentration of 2-OG was controlled within a certain range, GlnB protein could still bind to the α subunit to different degrees, but this interaction gradually weakened with the increase of 2-OG concentration (Figure 2C, lanes 2–4). When the concentration of 2-OG increased to 500 μM, the protein complex completely disassociated (Figure 2C, lanes 5–7). This indicated that the interaction between the GlnB protein and the α subunit was regulated by 2-OG, which is consistent with the regulation of its homologs in *E. coli* and *Synechocystis* sp. PCC 6803 (Gerhardt et al., 2015; Hauf et al., 2016).

### Effects of the Signal Transduction Protein GlnB on the Activity of ACC and PCC *in vitro*

**FIGURE 3:**
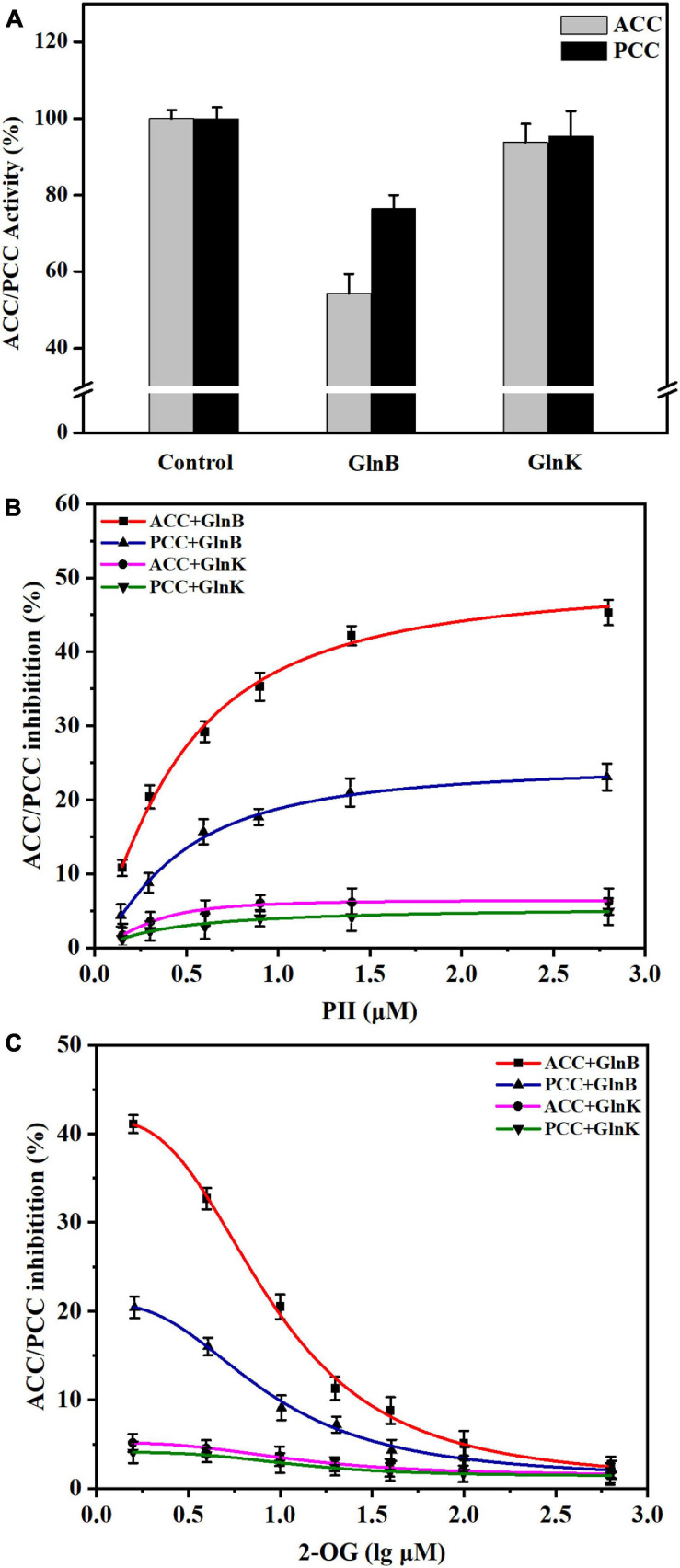
Influence of PII proteins and 2-OG on the activity of acetyl-CoA carboxylase (ACC) and propionyl-CoA carboxylase (PCC) *in vitro*. **(A)** The changes of ACC and PCC activity *in vitro* caused by the addition of GlnB or GlnK protein. The enzyme activity in the reaction system without PII proteins was defined as 100%. The enzyme activity in the reaction system containing 5 μM GlnB or GlnK protein was recorded for comparison. **(B)** The inhibition of ACC and PCC activity by PII proteins at different concentrations. The coupled enzyme activity assays were carried out in reaction systems without 2-OG. **(C)** Release of the inhibition of ACC and PCC by 2-OG at different concentrations. The coupled enzyme activity assays were performed in reaction systems including 3 μM PII proteins. All data represent the mean values of three independent technical replicates, and the error bars represent the standard deviations.

Considering that PCC had been reported to share the same α subunit with ACC in actinobacteria (Diacovich et al., 2002; Gago et al., 2006; Oh et al., 2006), the effects of the interaction between GlnB protein and the α subunit on the *in vitro* activity of ACC and PCC was assessed using coupled enzyme activity assays. Compared to the activity of ACC and PCC in the reaction system without GlnB protein, which was defined as 100%, the activity of ACC and PCC, respectively, decreased to 54.23 and 76.51% in the presence of GlnB protein (Figure 3A). This result confirmed that the interaction between GlnB protein and the α subunit could inhibit the activity of ACC and PCC. However, GlnK protein had almost no influence on the activity of these two enzymes, which was consistent with the co-precipitation results.

Further assays with different concentrations of PII protein showed that when the concentration of GlnB protein increased from 0 to 1 μM, the inhibition of ACC and PCC intensified sharply, and gradually stabilized at a GlnB protein concentration above 1.5 μM (Figure 3B). The maximal calculated inhibition of ACC by GlnB protein reached 50.04%, while the maximal calculated inhibition of PCC by GlnB protein reached only 24.69% (Figure 3B). This indicated that GlnB protein had a stronger inhibitory effect on ACC than on PCC. In order to analyze whether 2-OG could eliminate the inhibitory effects of GlnB protein on ACC and PCC, the coupled enzyme activity assays were carried out in the presence of different concentrations of 2-OG. The results showed that when the concentration of 2-OG increased from 0 to 20 μM, the inhibition of ACC and PCC was greatly alleviated, and when the concentration of 2-OG reached 500 μM, the inhibition of ACC and PCC was almost completely eliminated (Figure 3C). This demonstrated that the inhibition of ACC and PCC activity by GlnB protein could be alleviated by 2-OG in a concentration-dependent manner.

### Influence of PII Protein Deletion on the Carboxylation Reaction Catalyzed by ACC and PCC *in vivo*

**FIGURE 4:**
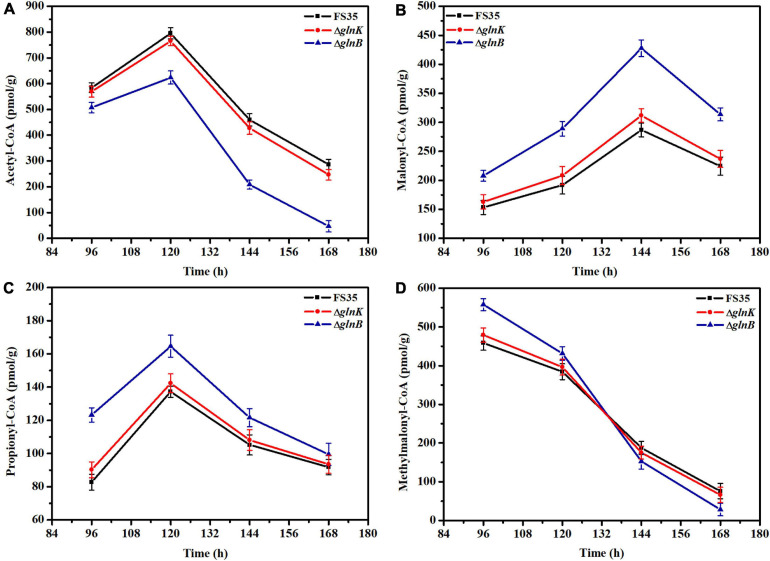
Changes in the intracellular concentrations of coenzyme A esters induced by the deletion of PII proteins. **(A)** Changes of intracellular acetyl-CoA concentration caused by the deletion of GlnB or GlnK. **(B)** Changes of intracellular malonyl-CoA concentration induced by the removal of GlnB or GlnK. **(C)** Changes of intracellular propionyl-CoA concentration caused by the deletion of GlnB or GlnK. **(D)** Changes of intracellular methylmalonyl-CoA concentration induced by the removal of GlnB or GlnK. The concentrations of coenzyme A esters in the parent strain FS35 were used as reference for comparison. The Δ*glnB* represents the *glnB* deletion strain based on the parent strain FS35. The Δ*glnK* represents the *glnK* deletion strain based on the parent strain FS35. All data represent the mean values of three independent technical replicates, and the error bars represent the standard deviations.

The effect of GlnB protein on the activity of ACC and PCC *in vivo* was assessed by measuring the changes in the intracellular concentrations of substrates (acetyl-CoA and propionyl-CoA) and products (malonyl-CoA and methylmalonyl-CoA) caused by the knockout of the *glnB* gene. As shown in Figure 4, during the stationary phase of fermentation (96–168 h), the amount of acetyl-CoA in the knockout strain Δ*glnB* was always lower than in the parental strain FS35, while the content of malonyl-CoA was always higher (Figures 4A,B). In detail, the content of acetyl-CoA in strain Δ*glnB* increased at a lower rate than in FS35 during 96–120 h, and then decreased at a faster rate in the subsequent fermentation period. However, the amount of malonyl-CoA in strain Δ*glnB* increased at a higher rate during 96–144 h (Figures 4A,B). These results illustrated that the removal of GlnB protein enhanced the conversion of acetyl-CoA into malonyl-CoA, indicating the release of ACC inhibition *in vivo*. However, the increase of malonyl-CoA concentration in strain Δ*glnB* during 120–144 h was lower than during 96–120 h, and the amount of malonyl-CoA decreased at a faster rate than in FS35 during 144–168 h (Figure 4B). These changes might be caused by the increased consumption of malonyl-CoA as a substrate for downstream biochemical reactions or as a precursor for the biosynthesis of FK520 in strain Δ*glnB* (Figure 1A).

Due to the increased supply of malonyl-CoA, the amount of propionyl-CoA in strain Δ*glnB* was always higher than in the parental strain FS35. In detail, the increase rate of propionyl-CoA in strain Δ*glnB* was lower than in FS35 during 96–120 h, and the decrease rate was higher after 120 h (Figure 4C). In addition, the content of methylmalonyl-CoA in strain Δ*glnB* was higher than in FS35 during the early stationary phase (Figure 4D). These results demonstrated that the removal of GlnB protein also increased the conversion of propionyl-CoA into methylmalonyl-CoA, indicating the release of PCC inhibition *in vivo*. The dramatic reduction of the methylmalonyl-CoA concentration in strain Δ*glnB* during the stationary phase, which was even faster than in the parental strain FS35, might be caused by the increased consumption of methylmalonyl-CoA as a precursor for the biosynthesis of FK520. In summary, the deletion of the *glnB* gene alleviated the inhibition of ACC and PCC activity by its encoded GlnB protein, and increased the supply of the precursors malonyl-CoA and methylmalonyl-CoA for the biosynthesis of FK520.

### GlnK Can Act Redundantly to Maintain Metabolic Homeostasis in the Absence of GlnB Protein

**FIGURE 5:**
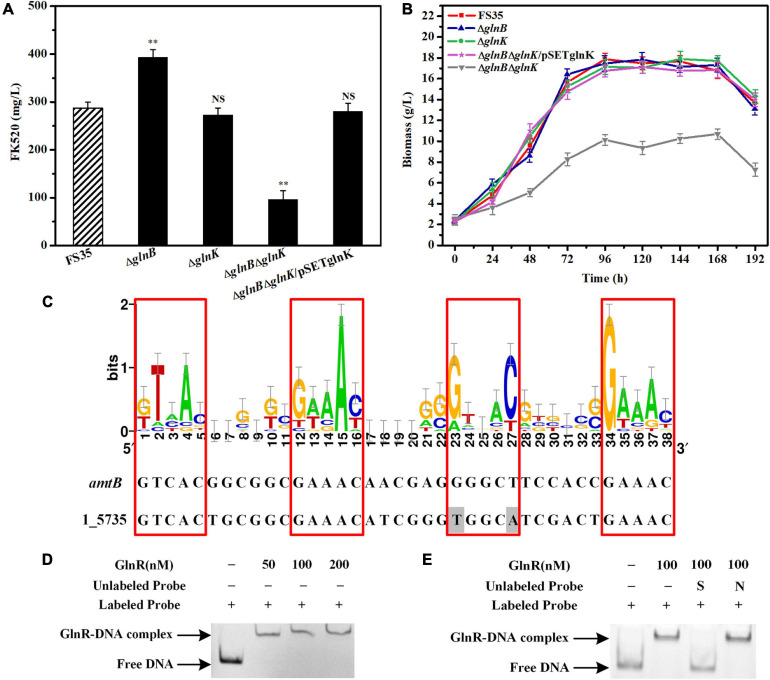
Effects of the PII proteins on the FK520 yield and biomass accumulation, and the regulation of *glnK* gene expression by GlnR. **(A)** The changes of FK520 yield caused by the deletion of PII proteins or the complementation of GlnK protein. The FK520 yield of the parent strain FS35 was used as a reference for comparison. **(B)** Changes of biomass caused by the deletion of PII protein or the complementation of GlnK protein. The biomass of the parent strain FS35 was used as a reference for comparison. **(C)** The DNA binding sites of the regulator GlnR in the promoter region of the *amtB-glnK-glnD* operon in *Streptomyces hygroscopicus* var. *ascomyceticus*. The sequence logo was created using WebLogo (http://weblogo.berkeley.edu/logo.cgi) based on the known DNA binding sites of GlnR in *Streptomyces coelicolor*. The binding site of GlnR in the promoter region of *amtB* from *S. coelicolor* was used as a reference for comparison. The amino acid sequences of the promoter region of 1_5735 and the GlnR regulator from *S. hygroscopicus* var. *ascomyceticus* have been uploaded to the GeneBank database (1_5735: MW936366, GlnR: MW936365). **(D)** EMSA for determining the formation of a protein-DNA complex between the GlnR regulator and the promoter sequence of the *amtB-glnK-glnD* operon. **(E)** Assessment of the specificity of the interaction between the GlnR regulator and the promoter of *amtB-glnK-glnD* operon. All blots on the gel map indicated the free DNA or GlnR-DNA complex fractionated by EMSA. *S* represents the specific unlabeled probe used for competitive binding to the regulator GlnR. *N* represents the non-specific unlabeled probe. The Δ*glnB* represents the *glnB* deletion strain based on the parent strain FS35. The Δ*glnK* represents the *glnK* deletion strain based on the parent strain FS35. The Δ*glnB*Δ*glnK* represents the *glnB*-*glnK* double deletion strain based on the parent strain FS35. The Δ*glnB*Δ*glnK*/pSETglnK represents the *glnK* complementation strain based on the *glnB*-*glnK* double deletion strain Δ*glnB*Δ*glnK*. All data represent the mean values of five independent technical replicates, and the error bars represent the standard deviations. *Asterisks* indicate significant differences between the parent strain FS35 and the engineering strains. *P*-values were calculated using two-tailed Student’s *t*-test. Double asterisk indicates *P* < 0.01. *NS* means the difference is not significant.

The fermentation results showed that the yield of FK520 in the knockout strain Δ*glnB* was significantly improved (to 390 ± 10 mg/L) due to the increased supply of the precursors malonyl-CoA and methylmalonyl-CoA. However, the yield of FK520 in the knockout strain Δ*glnK* was not significantly different from the parent strain FS35 (Figure 5A). The biomass measurement results showed that the individual deletion of *glnB* or *glnK* did not affect the biomass accumulation, while the double deletion of *glnB* and *glnK* resulted in a significant decrease of the biomass yield (Figure 5B). Furthermore, colonies of the *glnB*-*glnK* double mutant on agar plates also showed poorer mycelial growth (Supplementary Figure 4). At the same time, the yield of FK520 was also decreased sharply in the *glnB*-*glnK* double knockout strain (Figure 5A). These results indicated that the double deletion of *glnB* and *glnK* might cause a disturbance of intracellular metabolism. However, when GlnK expression was complemented in the *glnB*-*glnK* double deletion strain, the biomass accumulation and the FK520 production both recovered to the same levels as in the parental strain FS35 (Figures 5A,B). This suggested that the dysregulation caused by the removal of GlnB protein might be compensated by GlnK protein, which appears to have a partially redundant role.

However, unlike the constitutive expression of GlnB protein, the expression of GlnK protein is controlled by nitrogen regulator I (NRI) in *E. coli*, or by the transcriptional regulator GlnR in actinobacteria (vanHeeswijk et al., 1996; Fink et al., 2002; Amon et al., 2008). Therefore, to assess whether GlnK protein is also regulated by GlnR in *S. hygroscopicus* var. *ascomyceticus*, the known DNA binding sites of the GlnR regulator in *S. coelicolor* were collected and loaded into WebLogo^1^ to create a sequence logo (Figure 5C), and the binding site of the GlnR regulator in the promoter region of the *amtB-glnK-glnD* operon from *S. hygroscopicus* var. *ascomyceticus* was identified using the Motif Alignment and Search Tool (MAST) (Figure 5C). An EMSA with a His-labeled promoter probe and different concentrations of GlnR protein demonstrated that the GlnR regulator did bind to the promoter region of *amtB-glnK-glnD* operon (Figure 5D). The specificity of this binding was further confirmed by EMSA, in which specific and non-specific unlabeled proteins were added for the competitive binding (Figure 5E). These results indicated that when GlnB protein was absent, GlnK protein regulated the related metabolism under the control of the global regulator GlnR to maintain the normal growth of the strain. This was also demonstrated by the up-regulated expression of *glnK* in the *glnB* deletion strain Δ*glnB* compared with the parent strain FS35 (Supplementary Figure 5).

### Further Improvement of FK520 Yield by Combinatorial Genetic Manipulation

**FIGURE 6:**
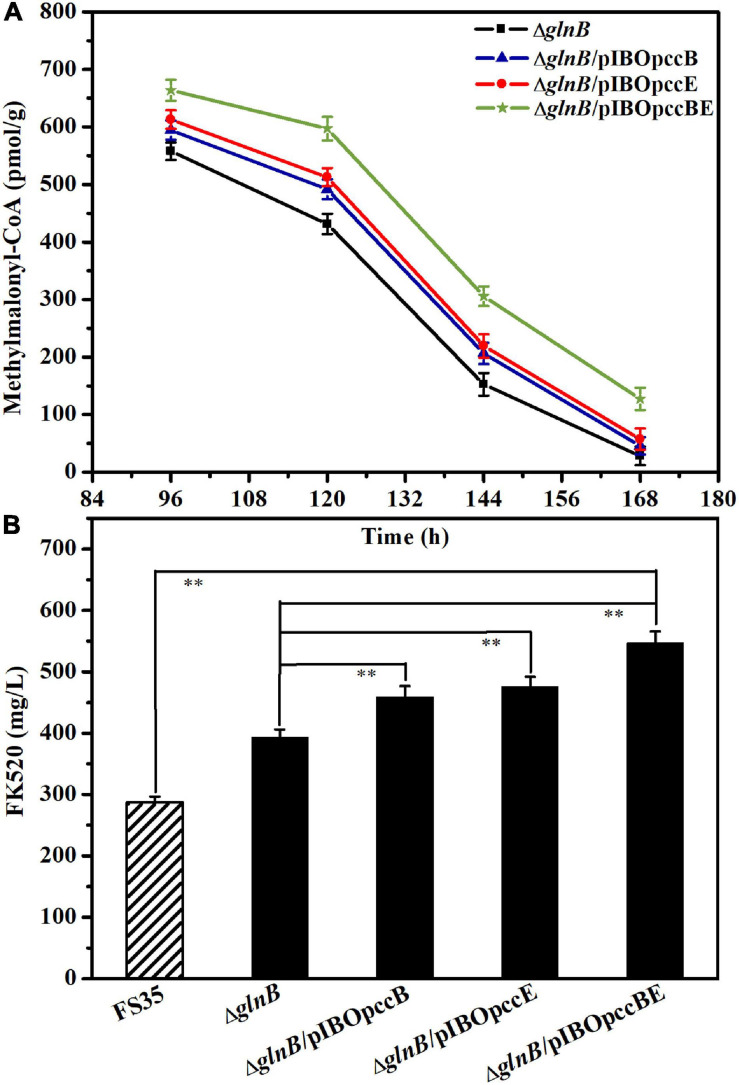
Influence of *pccB* and *pccE* overexpression on the methylmalonyl-CoA concentration and FK520 yield. **(A)** Changes of the methylmalonyl-CoA content caused by the overexpression of *pccB* and *pccE* alone or in combination. The concentration of methylmalonyl-CoA in the deletion strain Δ*glnB* was used as a reference for comparison. The data represent the mean values of three independent technical replicates, and the error bars represent the standard deviations. **(B)** Changes of the FK520 yield caused by the overexpression of *pccB* and *pccE* alone or in combination. The FK520 yield of the deletion strain Δ*glnB* and parent strain FS35 were used as references for comparison. The data represent the mean values of five independent technical replicates, and the error bars represent the standard deviations. *Asterisks* indicate significant differences between the parent strain FS35 and the engineering strains. *P*-values were calculated using two-tailed Student’s *t*-test. Double asterisk indicates *P* < 0.01. The Δ*glnB* represents the *glnB* deletion strain based on the parent strain FS35. The Δ*glnB*/pIBOpccB represents the *pccB* overexpression strain based on the Δ*glnB* strain. The Δ*glnB*/pIBOpccE represents the *pccE* overexpression strain based on the Δ*glnB* strain. The Δ*glnB*/pIBOpccBE represents the *pccB* and *pccE* co-overexpression strain based on the Δ*glnB* strain.

Although the removal of GlnB protein relieved its inhibition of ACC and PCC, increasing the yield of FK520 to a certain extent, the content of methylmalonyl-CoA in the late period of fermentation was still low (Figure 4D). Previous research suggested that the specific β subunit (responsible for substrate recognition) and ε subunit (responsible for protein- protein interactions) are crucial for the carboxylation activity of PCC (Diacovich et al., 2002; Demirev et al., 2011; Tong, 2013). The results of amino acid sequence alignment showed that the 1_3398 gene (*pccB*) encoded the β subunit, while the 1_3397 gene (*pccE*) encoded the ε subunit of PCC in *S. hygroscopicus* var. *ascomyceticus* (Supplementary Figure 6). Therefore, in order to further improve the production of FK520, the *pccB* and *pccE* genes were overexpressed alone or in combination in the *glnB* knockout strain to further increase the supply of methylmalonyl-CoA. The fermentation results showed that the single overexpression of *pccB* or *pccE* improved the supply of methylmalonyl-CoA during the early stationary phase (96–144 h), and increased the yield of FK520 to a certain extent (Figures 6A,B). Moreover, the co-overexpression of *pccB* and *pccE* in strain Δ*glnB* ensured a sufficient supply of methylmalonyl-CoA throughout the stationary phase (96–168 h), thus increasing the production of FK520 to 550 ± 20 mg/L, which was 1.9-fold higher than the yield of the parent strain FS35 (287 ± 9 mg/L) (Figures 6A,B).

FK523 is the main impurity in the production of FK520, which is formed by the assembly of methylmalonyl-CoA at the C21 position of the macrolide skeleton instead of the specific precursor ethylmalonyl-CoA (Yu et al., 2019). The quantification of the FK523 yield and the FK523/FK520 ratio in the parental strain FS35 and the co-overexpression strain Δ*glnB*/pIBOpccBE showed that although the FK523 yield of strain Δ*glnB*/pIBOpccBE was also increased, the FK523/FK520 ratio did not increased significantly (Supplementary Figure 7). This confirmed that the genetic engineering methods for increasing the yield of FK520 described in this study are reasonable and effective. Nonetheless, the reduction of the FK523 ratio requires further efforts in future studies.

## Discussion

As multifunctional signal integration regulators, the three types of PII family proteins play different roles in maintaining the homeostasis between carbon and nitrogen metabolism (Huergo et al., 2013; Esteves-Ferreira et al., 2018; Forchhammer and Selim, 2020). GlnK is mainly responsible for the regulation of *amtB*, the structural gene encoding an ammonia transporter (Zhang et al., 2006). Similarly, GlnB is mainly associated with the regulation of *glnA*, the GS structural gene (Schwarz et al., 2014), and NifI is related to the regulation of structural genes for nitrogenase (*nifH*, *nifD*, and *nifK*) (Arcondeguy et al., 2001). GlnK is widely distributed in a variety of microorganisms, GlnB is mainly found in Proteobacteria (Huergo et al., 2013), while NifI is present in the methanogenic archaea and some anaerobic bacteria (Zalutskaya et al., 2015). Before this study, only GlnK had been identified in actinobacteria (Arcondeguy et al., 2001; Burkovski, 2003). However, we demonstrated that both GlnB and GlnK are present in *S. hygroscopicus* var. *ascomyceticus*. To our best knowledge, this is the first study to demonstrate that two types of PII proteins exist simultaneously in actinobacteria, providing a new perspective on PII family signal transduction proteins, with possible implications for further research in other actinobacteria.

The GlnB and GlnK proteins from *S. hygroscopicus* var. *ascomyceticus* identified in this study showed 66.96% amino acid sequence identity. This was similar to the 67% sequence identity between GlnB and GlnK in *E. coli* (Xu et al., 1998). Previous studies had shown that the GlnB protein from *E. coli* could inhibit the activity of ACC by forming a ternary complex with its BC and BCCP subunits (Gerhardt et al., 2015). Although GlnK from *E. coli* can also bind to the BCCP subunit of ACC, it has no effect on ACC activity because it does no interaction with the BC-BCCP complex (Broussard et al., 2013; Rodrigues et al., 2014). This study demonstrated that GlnB from *S. hygroscopicus* var. *ascomyceticus* could inhibit the activity of ACC by binding to the α subunit (a fusion protein corresponding to the BC and BCCP subunits), while GlnK did not interact with the α subunit of ACC and had no effect on its activity. Previous studies have also shown that GlnB from *A. brasilense* could inhibit the activity of ACC by binding to the BC-BCCP complex, but GlnK could not (Gerhardt et al., 2015). These all indicate that in the PII family of signal transduction proteins, only GlnB can specifically interact with the subunits of ACC and regulate its activity.

In contrast to the ACC of bacteria such as *E. coli*, which consists of a BC subunit, a BCCP subunit and two CT subunits (Cronan and Waldrop, 2002), or the ACC of most eukaryotic organisms, which is a fusion protein containing functional domains corresponding to BC, BCCP, and CT (Tong, 2013), the ACC of *S. hygroscopicus* var. *ascomyceticus* is composed of an α subunit comprising BC and BCCP functional domains, and a β subunit corresponding to CT (Arabolaza et al., 2010). Moreover, in actinomycetes such as *M. tuberculosis* and *S. coelicolor*, ACC shares the same α subunit with PCC (Diacovich et al., 2002; Demirev et al., 2011). On this basis, we demonstrated for the first time that GlnB could not only regulate the activity of ACC, but also affect the activity of PCC in *S. hygroscopicus* var. *ascomyceticus*. Previous studies only showed the regulation of ACC activity by PII proteins, while this paper expands the regulatory range, uncovering a new regulatory target of PII proteins.

Lipids, which are synthesized with malonyl-CoA as the starting unit, are important carbon storage compounds for plants and microalgae, such as *A. thaliana* and *C. reinhardtii* (Forchhammer and Selim, 2020). It had been demonstrated that GlnB from *A. thaliana* can regulate the activity of ACC by interacting with its subunits, to control the synthesis of fatty acids (Bourrellier et al., 2010). It was also demonstrated that the deletion of PII protein in *C. reinhardtii* resulted in the dysregulation of ACC activity, significantly increasing the accumulation of lipids (Zalutskaya et al., 2015). In actinomycetes such as *S. coelicolor* and *S. hygroscopicus* var. *ascomyceticus*, the biosynthesis of malonyl-CoA catalyzed by ACC and methylmalonyl-CoA catalyzed by PCC is crucial for the production of antibiotics (Rodriguez et al., 2001; Song et al., 2017). To our best knowledge, no previous studies investigated the effect of PII proteins on the synthesis of antibiotics. Here, we demonstrated for the first time that the deletion of GlnB could significantly increase the yield of FK520 by improving the supply of the precursors malonyl-CoA and methylmalonyl-CoA, providing a new strategy for promoting the production of antibiotics in other actinobacteria.

To maintain the intracellular homeostasis of carbon and nitrogen metabolism, GlnB and GlnK proteins perform their respective major regulatory functions in a focused manner when both are present at the same time (Huergo et al., 2013). However, these two proteins are also partially redundant in their regulatory functions. When either protein is missing, the other protein will compensate for its function to maintain the balance of intracellular biochemical reactions (vanHeeswijk et al., 1996; Yurgel et al., 2010; Gosztolai et al., 2017). In this study, the single deletion of *glnB* in *S. hygroscopicus* var. *ascomyceticus* did not affect the accumulation of biomass, and the complementation of GlnK in the double deletion strain Δ*glnB*Δ*glnK* restored normal strain growth. These finding indicated that in *S. hygroscopicus* var. *ascomyceticus*, GlnK could compensate for the lack of GlnB to regulate the corresponding metabolism and maintain basic physiological activities, which was also found in other microorganisms.

Even if there are functional overlaps between GlnB and GlnK, the conditions under which they exert their respective regulatory functions are not completely identical. For example, both GlnB and GlnK can regulate the activity of GS following uridylylation or de-uridylylation by GlnD (Rodrigues et al., 2014; Gerhardt et al., 2015), and the expression of GlnB is constitutive (vanHeeswijk et al., 1996), while the expression of GlnK can be activated by the global nitrogen regulators GlnR or NRI under nitrogen starvation (vanHeeswijk et al., 1996; Fink et al., 2002; Amon et al., 2008). This study demonstrated that the global nitrogen regulator GlnR from *S. hygroscopicus* var. *ascomyceticus* could specifically bind to the promoter region of the *amtB-glnK-glnD* operon. This indicated that the expression of GlnK in *S. hygroscopicus* var. *ascomyceticus* might also be regulated by GlnR, similar to other actinobacteria. The detailed mechanism through which the global nitrogen regulator GlnR affects the expression of GlnK merits further investigation in future studies.

Taken together, this study demonstrates for the first time that the GlnB signal transduction protein inhibits the activity of ACC and PCC in *S. hygroscopicus* var. *ascomyceticus*. The knockout of *glnB* enhanced the supply of malonyl-CoA and methylmalonyl-CoA by relieving the inhibition of ACC and PCC, thereby improving the production of FK520 to 390 ± 10 mg/L. On this basis, the co-overexpression of the β and ε subunits of PCC further increased the FK520 yield to 550 ± 20 mg/L, which was 1.9-fold higher than that the of parent strain FS35 (287 ± 9 mg/L). The successful improvement of the FK520 yield by eliminating the inhibition of ACC and PCC activity by GlnB protein provides a reference for increasing the production of other antibiotics.

## Data Availability Statement

The datasets presented in this study can be found in online repositories. The names of the repository/repositories and accession number(s) can be found in the article/Supplementary Material.

## Author Contributions

PW carried out the experimental work, analyzed the data, and wrote the manuscript. XW helped in analyzing the data. YY helped in the experimental analysis. MH helped in performing the experiments. WT helped in revising the manuscript. WG helped in writing the manuscript. JW designed the experiments and supervised the work. All authors read and approved the manuscript.

## Conflict of Interest

The authors declare that the research was conducted in the absence of any commercial or financial relationships that could be construed as a potential conflict of interest.

## Footnotes

1 http://weblogo.berkeley.edu/logo.cgi

## Abbreviations

FK520: Ascomycin
ACC: Acetyl-CoA carboxylase
PCC: Propionyl-CoA carboxylase
BC: Biotin carboxylase
BCCP: Biotin carboxyl carrier protein
CT: Carboxyl transferase
2-OG: 2-oxoglutarate
ATP: Adenosine triphosphate
ADP: Adenosine diphosphate
GS: Glutamine synthetase
NAGK: N-acetyl-l-glutamate kinase
LB: Luria-Bertani medium
GEO: Gene Expression Omnibus database
NCBI: National Center for Biotechnology Information database
FK523: A by-product of FK520 biosynthesis
IPTG: Isopropyl β-d-thiogalactoside
SDS-PAGE: Sodium dodecyl sulfate-polyacrylamide gel electrophoresis
EMSA: Electrophoretic mobility shift assays
PK: Pyruvate kinase
LDH: Lactate dehydrogenase
NADH: Nicotinamide adenine dinucleotide (reduced)
DTT: Dithiothreitol
UPLC: Ultra-high performance liquid chromatography
ESI: Electrospray ionization
MS: Mass spectrometry
EDTA: Ethylenediaminetetraacetic acid
TBE: Tris borate EDTA
SD: Standard deviations
NRI: Nitrogen regulator I.
