# Supplementary material for: Increasing the Ascomycin Yield by Relieving the Inhibition of Acetyl/Propionyl-CoA Carboxylase by the Signal Transduction Protein GlnB
Source: Front Microbiol. 2021 May 26;12:684193. doi: 10.3389/fmicb.2021.684193 (PMC8187598; doi:10.3389/fmicb.2021.684193)
Supplement: Supplementary file 1 [file Data_Sheet_1.docx]

Supplementary Material

# Supplementary Tables

**Table 1** Strains and plasmids used in this study

| **Strains/plasmids** | **Descriptions** | **source** |
| --- | --- | --- |
| *S. hygroscopicus* var. *ascomyceticus* | | |
| FS35 | Mutant derived from ATCC 14891 | ([Qi et al., 2012](#_ENREF_4)) |
| Δ*glnB* | *glnB* deletion strain based on FS35 | This study |
| Δ*glnK* | *glnK* deletion strain based on FS35 | This study |
| Δ*glnB*Δ*glnK* | *glnB* and *glnK* double deletion strain based on FS35 | This study |
| Δ*glnB*Δ*glnK*/pSETglnK | *glnK* deletion strain based on Δ*glnB*Δ*glnK* | This study |
| Δ*glnB*/pIBOpccB | *pccB* overexpression strain based on Δ*glnB* | This study |
| Δ*glnB*/pIBOpccE | *pccE* overexpression strain based on Δ*glnB* | This study |
| Δ*glnB*/pIBOpccBE | *pccB* and *pccE* co- overexpression strain based on Δ*glnB* | This study |
| *E.coli* | | |
| DH5α | Plasmid construction and general cloning | Novagen, USA |
| ET12567/pUZ8002 | Plasmid donor for intergeneric conjugation, Cm^R^, Kan^R^ | ([Kieser et al., 2000](#_ENREF_2)) |
| BL21(DE3) | Host for protein overexpression | Novagen, USA |
| Plasmids | | |
| pET28a(+) | Vector for protein expression, Kan^R^ | Novagen, USA |
| pKC1139 | Temperature-sensitive shuttle vector for gene deletion, Apr^R^ | ([Huang et al., 2013](#_ENREF_1)) |
| pSET152 | Integrative shuttle vector, Amp^R^ | ([Huang et al., 2013](#_ENREF_1)) |
| pIB139 | pSET152-derived vector containing *ermE*p*, Apr^R^ | ([Qi et al., 2017](#_ENREF_3)) |
| pETglnB | *glnB* expression vector based on pET28a(+), Kan^R^ | This study |
| pETHglnB | *glnB* expression vector based on pET28a(+), contain a 6ⅹHis tag at N-terminal, Kan^R^ | This study |
| pETglnK | *glnK* expression vector based on pET28a(+), Kan^R^ | This study |
| pETaccA | *accA* expression vector based on pET28a(+), Kan^R^ | This study |
| pETHaccA | *accA* expression vector based on pET28a(+), contain a 6ⅹHis tag at N-terminal, Kan^R^ | This study |
| pETHglnR | *glnR* expression vector based on pET28a(+), contain a 6ⅹHis tag at N-terminal, Kan^R^ | This study |
| pKCglnB | *glnB* deletion vector based on pKC1139, Apr^R^ | This study |
| pKCglnK | *glnK* deletion vector based on pKC1139, Apr^R^ | This study |
| pSETglnK | *glnK* complemented vector based on pSET152, Amp^R^ |  |
| pIBOpccB | *pccB* overexpression vector based on pIB139, Apr^R^ | This study |
| pIBOpccE | *pccE* overexpression vector based on pIB139, Apr^R^ | This study |
| pIBOpccBE | *pccBE* overexpression vector based on pIB139, Apr^R^ | This study |

**Table 2** Primers used for gene deletion, complementation, and overexpression

| **Primers** | **Sequence (5’-3’)** | **Application** |
| --- | --- | --- |
| DglnB-UF | CCCAAGCTTCTGGTCATCGCCACCGGCGTCCGC(*Hin*dIII) | Knockout of gene *glnB* |
| DglnB-UR | CAGGACCCCGCTCGGATGACCGCGATAACGGCGACAAC |  |
| DglnB-DF | GTTGTCGCCGTTATCGCGGTCATCCGAGCGGGGTCCTG |  |
| DglnB-DR | GCTCTAGAGACCTTCCAGCTCACCTTCGCGATCCTCAC(*Xba*I) |  |
| VDglnB-F | CAACGGCCTGGAGTGCGACGAGTAC | Verification of *glnB* deletion |
| VDglnB-R | GACTGGTGGCCATCACCCCGTCCTG |  |
| DglnK-UF | CCCAAGCTTCTGGCCTTCGCGTTCTTCCAGCTGA(*Hin*dIII) | Knockout of gene *glnK* |
| DglnK-UR | GTTGTCCGTGGCCCTGTGGGCGTCCACCTTCTTGTTGTG |  |
| DglnK-DF | CACAACAAGAAGGTGGACGCCCACAGGGCCACGGACAAC |  |
| DglnK-DR | GCTCTAGACTTCCGGCACCCCGTCCGGCGGTGC(*Xba*I) |  |
| VDglnK-F | GTGTCGCGATGCTCGGCTGGCTCGC | Verification of *glnK* deletion |
| VDglnK-R | GCCCCGGGGCCTGGGCCCGCCAGTC |  |
| CglnK-PF | CGGGATCCGCGGTCCCCGCGGTGGTTGC(*Bam*HI) | Complementation of gene *glnK* |
| CglnK-PR | CCGCGGTGATGAGCTTCACTGTGGACGTCTCCTCGAAAC |  |
| CglnK-KF | GTTTCGAGGAGACGTCCACAGTGAAGCTCATCACCGCGG |  |
| CglnK-KR | GCTCTAGATCAGAGGGCGTCCGGACCGCGCTCG(*Xba*I) |  |
| OpccB-F | GGAATTCCATATGATGGCCGAGCCGGAGATCGC(*Nde*I) | Overexpression of gene *pccB* |
| OpccB-R | GCTCTAGACTAGAGGGGGATGTTGCCGTGCTTC(*Xba*I) |  |
| OpccE-F | GGAATTCCATATGATGACGCCTTCTGACACCTC(*Nde*I) | Overexpression of gene *pccE* |
| OpccE-R | GCTCTAGATCAATCCCCCCTCGGTACGGAG(*Xba*I) |  |
| OpccBE-BF | GGAATTCCATATGATGGCCGAGCCGGAGATCGC(*Nde*I) | Co-overexpression of genes *pccB* and *pccE* |
| OpccBE-BR | GTGTCAGAAGGCGTCATTGGATCCTCTAGAGGGGGATGTTG(RBS) |  |
| OpccBE-EF | CAACATCCCCCTCTAGAGGATCCAATGACGCCTTCTGACAC(RBS) |  |
| OpccBE-ER | GCTCTAGATCAATCCCCCCTCGGTACGGAGCG(*Xba*I) |  |
| pSET152-F | TGTAAAACGACGGCCAGT | Verification of Complementation mutant |
| pSET152-R | CAGGAAACAGCTATGAC |  |
| pIB139-F | CGATGCTGTTGTGGGCACA | Verification of overexpression mutant |
| pIB139-R | CGCGTTGGCCGATTCAT |  |

**Table 3** Primers used for protein expression, EMSA and qRT-PCR

| **Primers** | **Sequence (5’-3’)** | **Application** |
| --- | --- | --- |
| GlnB-F | CATGCCATGGATGAAGCTGATCACGGCCATCATCA(*Nco*I) | Expression of native protein GlnB |
| GlnB-R | CGGGATCCTCAGATGGCGTCCGGGCCGCGTTC(*Bam*HI) |  |
| GlnB-HF | GGAATTCCATATGAAGCTGATCACGGCCATCATCA(*Nde*I) | Expression of His-tagged protein GlnB |
| GlnB-HR | CGGGATCCTCAGATGGCGTCCGGGCCGCGTTC(*Bam*HI) |  |
| GlnK-F | CATGCCATGGATGAAGCTCATCACCGCGGTAGTGA(*Nco*I) | Expression of native protein GlnK |
| GlnK-R | CGGGATCCTCAGAGGGCGTCCGGACCGCGCTCG(*Bam*HI) |  |
| AccA-F | CATGCCATGGTGCGCAAGGTGCTCATCG(*Nco*I) | Expression of native α subunit of ACC |
| AccA-R | CGGGATCCTCAGTCCTTGATTTCGCAGATGAC(*Bam*HI) |  |
| AccA-HF | GGAATTCCATATGCGCAAGGTGCTCATCG(*Nde*I) | Expression of His-tagged α subunit of ACC |
| AccA-HR | CGGGATCCTCAGTCCTTGATTTCGCAGATGAC(*Bam*HI) |  |
| GlnR-HF | GGAATTCCATATGAGTTCCCTCCTGC(*Nde*I) | Expression of His-tagged protein GlnR |
| GlnR-HR | CGGGATCCCTAGCTGTTGGCCGGTC(*Bam*HI) |  |
| pBKD-F | AGCCAGTGGCGATAAGGCGGTCCCCGCGGTGGTTGC | Amplification for promoter of *amtB-glnK-glnD* operon |
| pBKD-R | AGCCAGTGGCGATAAGTGTGGACGTCTCCTCGAAACGAC |  |
| qRT-KF | ACCGAGGCCAGCGGCTAC | qRT-PCR analysis of gene *glnK* |
| qRT-KR | GCTCCAGACCTTGCCGTC |  |

# References

Huang, D., Li, S. S., Xia, M. L., Wen, J. P., and Jia, X. Q. (2013). Genome scale metabolic network guided engineering of *Streptomyces tsukubaensis* for FK506 production improvement. *Microb. Cell Fact.* 12**,** 52. doi: 10.1186/1475-2859-12-52.

Kieser, T., Bibb, M., Buttner, M., and Chater, K. (2000). Practical *Streptomyces* genetics. Norwich: The John Innes Foundation.

Qi, H. S., Lv, M. M., Song, K. J., and Wen, J. P. (2017). Integration of parallel C-13-labeling experiments and in silico pathway analysis for enhanced production of ascomycin. *Biotechnol. Bioeng.* 114**,** 1036-1044. doi: 10.1002/bit.26223.

Qi, H. S., Xin, X., Li, S. S., Wen, J. P., Chen, Y. l., and Jia, X. Q. (2012). Higher-level production of ascomycin (FK520) by *Streptomyces hygroscopicus* var. *ascomyceticus* irradiated by femtosecond laser. *Biotechnol. Bioprocess Eng.* 17**,** 770-779. doi: 10.1007/s12257-012-0114-2.
