# Supplementary material for: Increasing the Ascomycin Yield by Relieving the Inhibition of Acetyl/Propionyl-CoA Carboxylase by the Signal Transduction Protein GlnB
Source: Front Microbiol. 2021 May 26;12:684193. doi: 10.3389/fmicb.2021.684193 (PMC8187598; doi:10.3389/fmicb.2021.684193)
Supplement: Supplementary file 2 [file Data_Sheet_2.docx]

Supplementary Material

# Supplementary Figures

**Supplementary Figure 1**


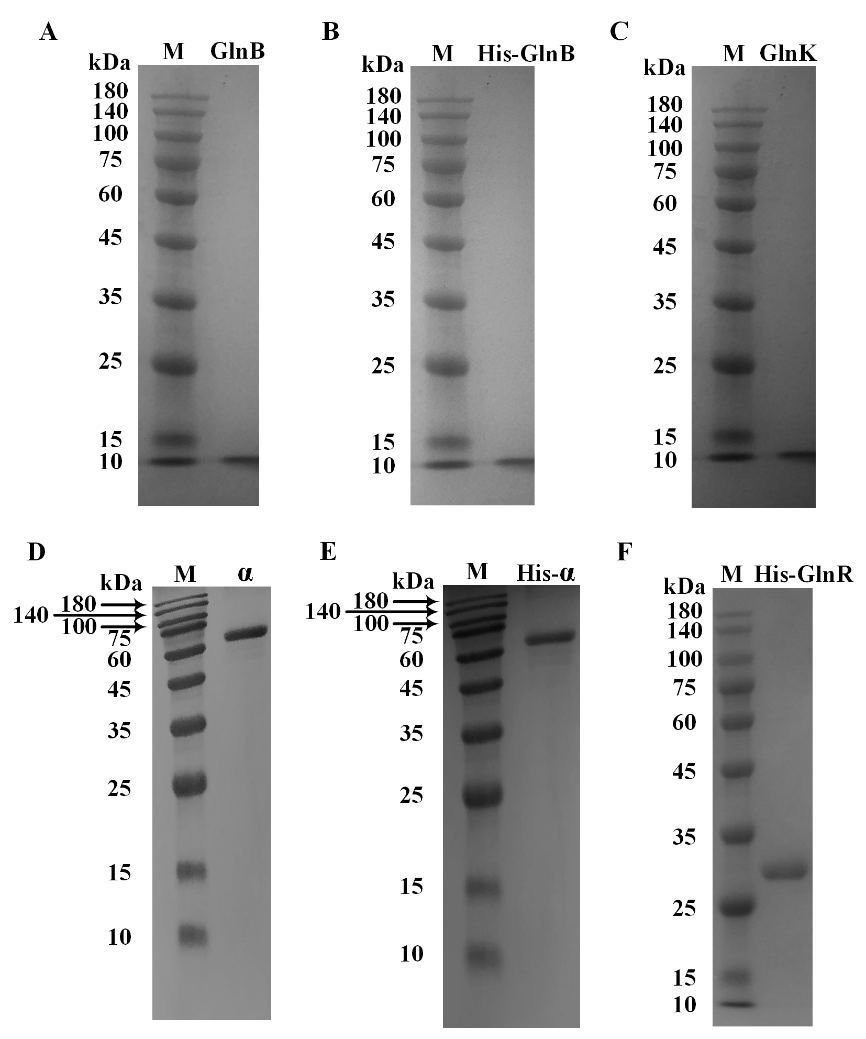


**Supplementary Figure 1.** SDS-PAGE profiles of the proteins used for EMSA and pull-down assays. **(A)** SDS-PAGE profile of the GlnB protein used for pull-down assays. **(B)** SDS-PAGE profile of the His-GlnB protein used for pull-down assays. **(C)** SDS-PAGE profile of the GlnK protein used for pull-down assays. **(D)** SDS-PAGE profile of the α subunit of ACC used for pull-down assays. **(E)** SDS-PAGE profile of the His-α subunit used for pull-down assays. **(F)** SDS-PAGE profile of the GlnR protein used for EMSA assays.

**Supplementary Figure 2**


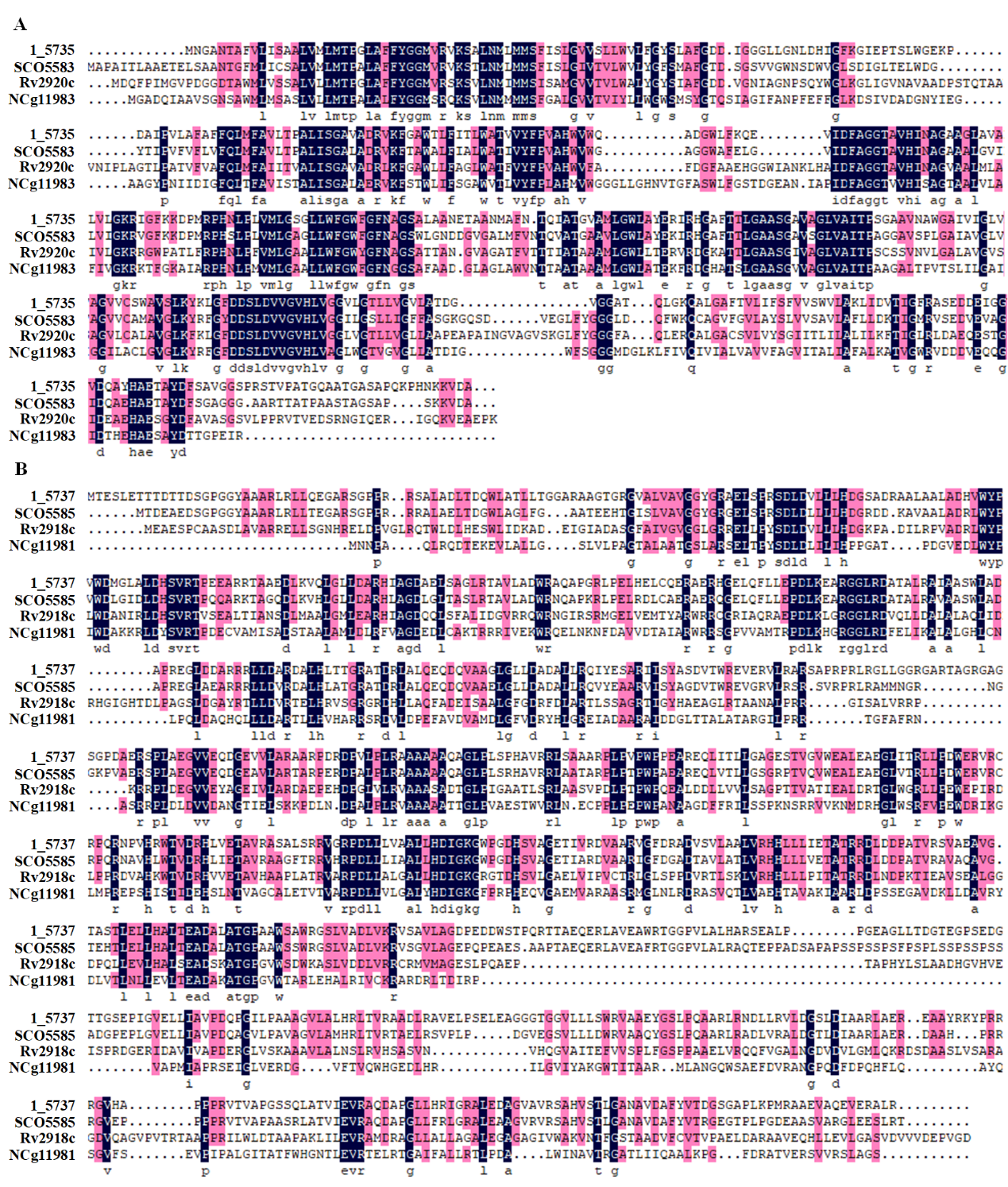


**Supplementary Figure 2.** Amino acid sequence alignment of AmtB and GlnD between *Streptomyces hygroscopicus* var. *ascomyceticus* and other actinobacteria. **(A)** Amino acid sequence alignment of AmtB between *S. hygroscopicus* var. *ascomyceticus* and other actinobacteria. **(B)** Amino acid sequence alignment of GlnD between *S. hygroscopicus* var. *ascomyceticus* and other actinobacteria. 1_5735 and 1_5737 respectively represent the AmtB and GlnD of *S. hygroscopicus* var. *ascomyceticus* FS35, and their amino acid sequences have been uploaded to the GeneBank database (1_5735: MW936367, 1_5737: MW936368). SCO5583 and SCO5585 respectively represent the AmtB and GlnD of *Streptomyces coelicolor* A3(2), which are retrieved from the NCBI database. Rv2920c and Rv2918c respectively represent the AmtB and GlnD of *Mycobacterium tuberculosis* H37Rv, which are retrieved from the NCBI database. NCg11983 and NCg11981 respectively represent the AmtB and GlnD of *Corynebacterium glutamicum* ATCC13032, which are retrieved from the NCBI database. The dark blue shadows highlight the identical residues and the residues with more than 75% sequence similarity are shaded in pink.

**Supplementary Figure 3**

**
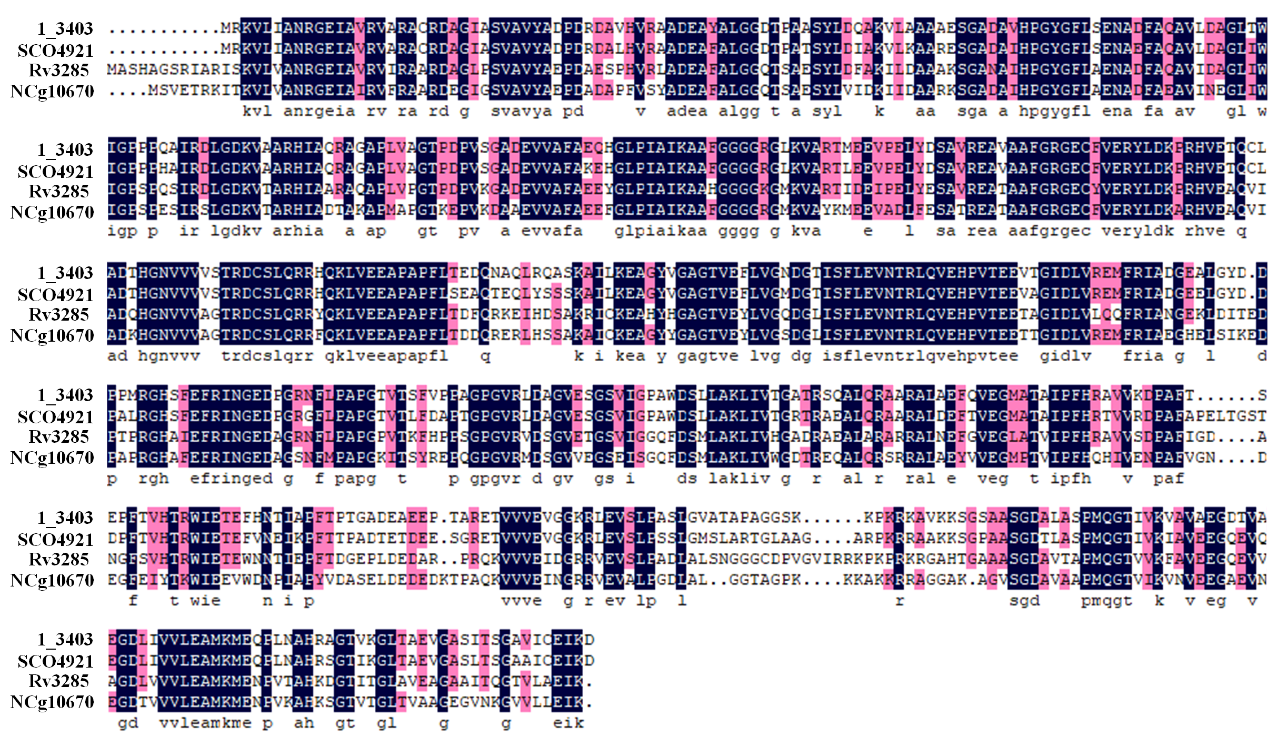
**

**Supplementary Figure 3.** Amino acid sequence alignment of the α subunit of ACC between *Streptomyces hygroscopicus* var. *ascomyceticus* and other actinobacteria. 1_3403 represents the α subunit of ACC from *S. hygroscopicus* var. *ascomyceticus* FS35, and its amino acid sequences has been uploaded to the GeneBank database (MW936361). SCO4921 represents the α subunit of ACC from *Streptomyces coelicolor* A3(2), which is retrieved from the NCBI database. Rv3285 represents the α subunit of ACC from *Mycobacterium tuberculosis* H37Rv, which is retrieved from the NCBI database. NCg10670 represents the α subunit of ACC from *Corynebacterium glutamicum* ATCC13032, which is retrieved from the NCBI database. The dark blue shadows highlight the identical residues and the residues with more than 75% sequence similarity are shaded in pink.

**Supplementary Figure 4**


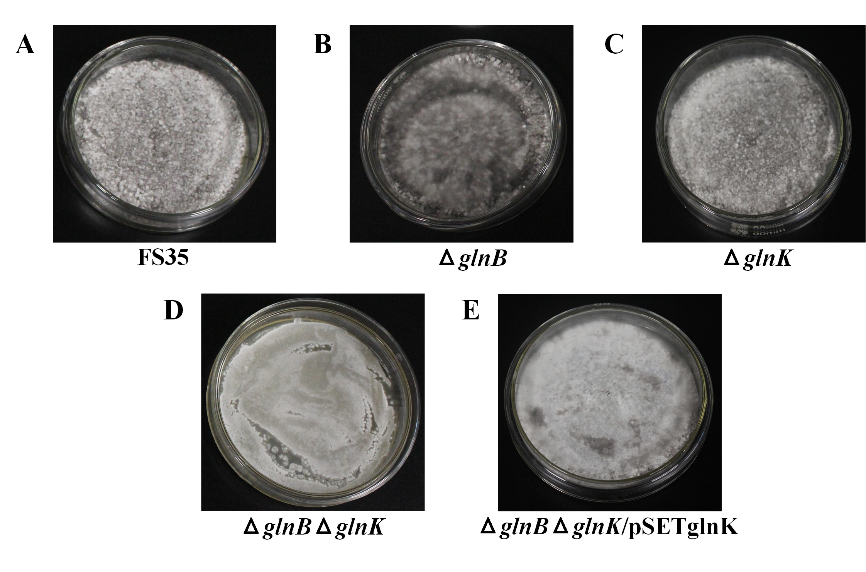


**Supplementary Figure 4.** Growth states of different strains on the flat plate after 20 days of culture. **(A)** Growth states of the parent strain FS35. **(B)** Growth states of the *glnB* deletion strain Δ*glnB*. **(C)** Growth states of the *glnK* knockout strain Δ*glnK*. **(D)** Growth states of the *glnB*-*glnK* double inactivation strain Δ*glnB*Δ*glnK*. **(E)** Growth states of the *glnK* complementation strain Δ*glnB*Δ*glnK*/pSETglnK.

**Supplementary Figure 5**


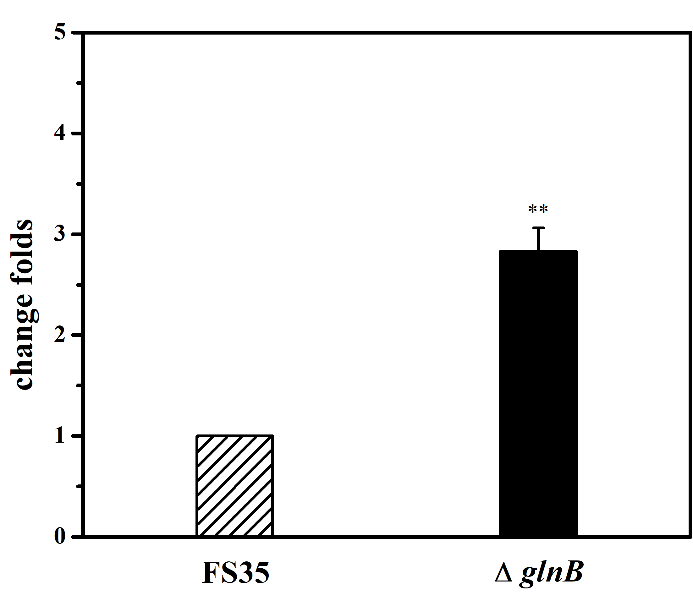


**Supplementary Figure 5.** Comparative transcriptional level analysis of *glnK* in the parent strain FS35 and the *glnB* deletion strain Δ*glnB*. The transcriptional level of *glnK* in the strain FS35 was defined as 1 and used as the control. All data represent the mean values of three independent technical replicates, and the error bars represent the standard deviations. *Asterisks* indicate significant differences between the parent strain FS35 and the engineering strains. *P* values were calculated using two-tailed Student’s *t* test. Double asterisk indicates *P* < 0.01.

**Supplementary Figure 6**


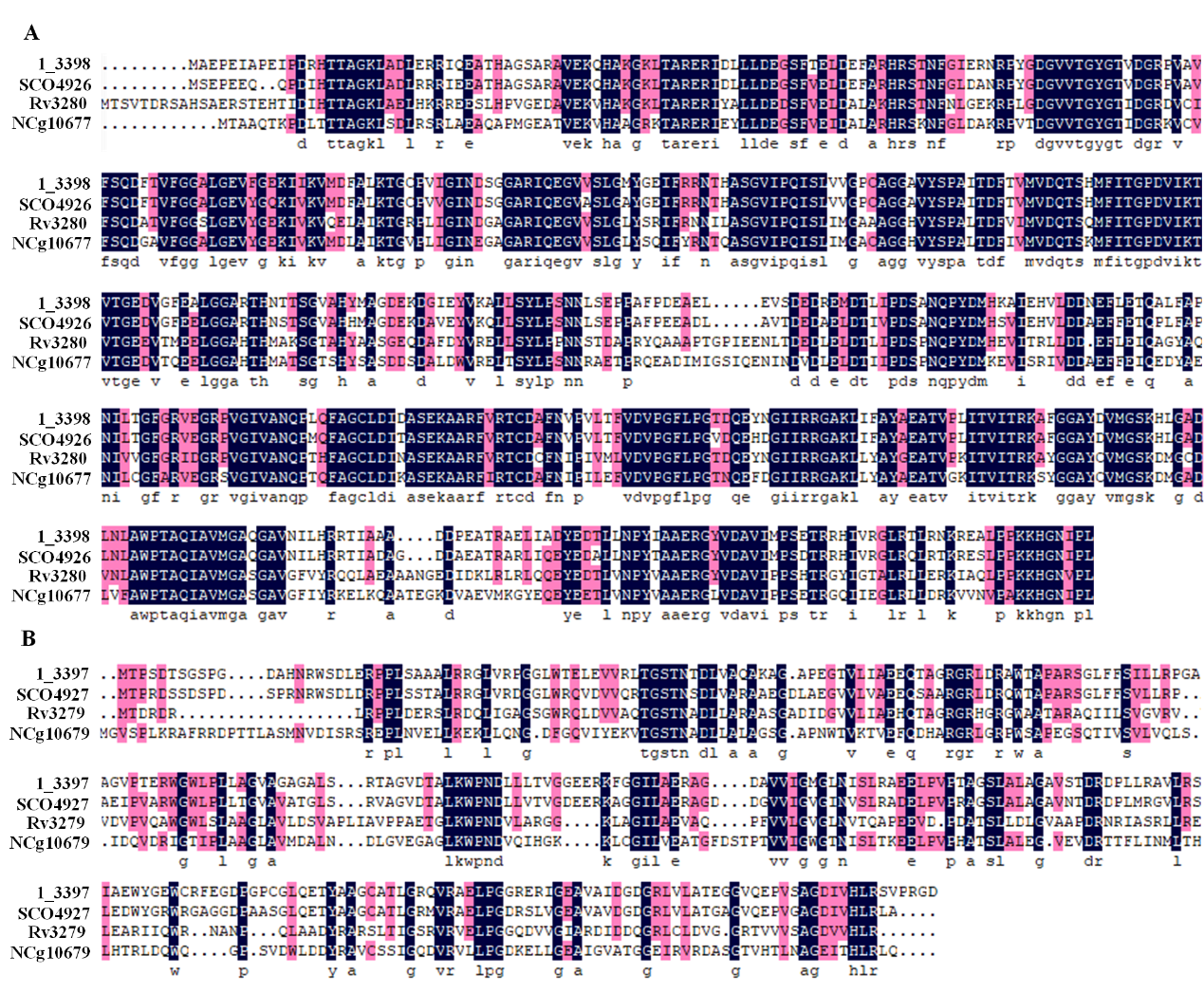


**Supplementary Figure 6.** Amino acid sequence alignment of PccB and PccE between *Streptomyces hygroscopicus* var. *ascomyceticus* and other actinobacteria. **(A)** Amino acid sequence alignment of PccB between *S. hygroscopicus* var. *ascomyceticus* and other actinobacteria. **(B)** Amino acid sequence alignment of PccE between *S. hygroscopicus* var. *ascomyceticus* and other actinobacteria. 1_3398 and 1_3397 respectively represent the PccB and PccE of *S. hygroscopicus* var. *ascomyceticus* FS35, and their amino acid sequences have been uploaded to the GeneBank database (1_3398: MW936363, 1_3397: MW936364). SCO4926 and SCO4927 respectively represent the PccB and PccE of *Streptomyces coelicolor* A3(2), which are retrieved from the NCBI database. Rv3280 and Rv3279 respectively represent the PccB and PccE of *Mycobacterium tuberculosis* H37Rv, which are retrieved from the NCBI database. NCg10677 and NCg10679 respectively represent the PccB and PccE of *Corynebacterium glutamicum* ATCC13032, which are retrieved from the NCBI database. The dark blue shadows highlight the identical residues and the residues with more than 75% sequence similarity are shaded in pink.

**Supplementary Figure 7**


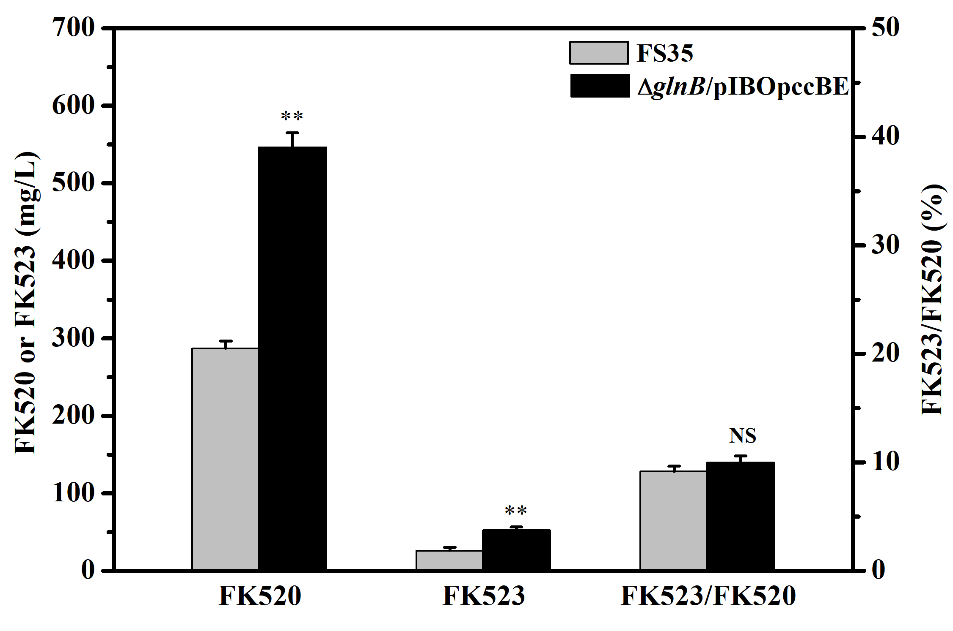


**Supplementary Figure 7.** Effect of genetic modification on the production of by-product FK523. All data represent the mean values of five independent technical replicates, and the error bars represent the standard deviations. *Asterisks* indicate significant differences between the parent strain FS35 and the engineering strains. *P* values were calculated using two-tailed Student’s *t* test. Double asterisk indicates *P* < 0.01. *NS* means the difference is not significant.
